# The 27th annual Nucleic Acids Research database issue and molecular biology database collection

**DOI:** 10.1093/nar/gkz1161

**Published:** 2019-12-22

**Authors:** Daniel J Rigden, Xosé M Fernández

**Affiliations:** 1 Institute of Integrative Biology, University of Liverpool, Crown Street, Liverpool L69 7ZB, UK; 2 Institut Curie, 25 rue d’Ulm, 75005 Paris, France

## Abstract

The 2020 Nucleic Acids Research Database Issue contains 148 papers spanning molecular biology. They include 59 papers reporting on new databases and 79 covering recent changes to resources previously published in the issue. A further ten papers are updates on databases most recently published elsewhere. This issue contains three breakthrough articles: AntiBodies Chemically Defined (ABCD) curates antibody sequences and their cognate antigens; SCOP returns with a new schema and breaks away from a purely hierarchical structure; while the new Alliance of Genome Resources brings together a number of Model Organism databases to pool knowledge and tools. Major returning nucleic acid databases include miRDB and miRTarBase. Databases for protein sequence analysis include CDD, DisProt and ELM, alongside no fewer than four newcomers covering proteins involved in liquid–liquid phase separation. In metabolism and signaling, Pathway Commons, Reactome and Metabolights all contribute papers. PATRIC and MicroScope update in microbial genomes while human and model organism genomics resources include Ensembl, Ensembl genomes and UCSC Genome Browser. Immune-related proteins are covered by updates from IPD-IMGT/HLA and AFND, as well as newcomers VDJbase and OGRDB. Drug design is catered for by updates from the IUPHAR/BPS Guide to Pharmacology and the Therapeutic Target Database. The entire Database Issue is freely available online on the Nucleic Acids Research website (https://academic.oup.com/nar). The NAR online Molecular Biology Database Collection has been revised, updating 305 entries, adding 65 new resources and eliminating 125 discontinued URLs; so bringing the current total to 1637 databases. It is available at http://www.oxfordjournals.org/nar/database/c/.

## NEW AND UPDATED DATABASES

The year 2020 sees the Nucleic Acids Research Database Issue reach its 27th annual issue. As usual, the 148 papers included span the full range of biological research. This year there are papers on 59 new databases (Table 1) while 79 resources provide Update papers covering recent developments. A further 10 papers cover updates of databases most recently published elsewhere (Table 2). The issue begins with reports from the major database providers at the U.S. National Center for Biotechnology Information (NCBI), the European Bioinformatics Institute (EBI) and the National Genomics Data Center (NGDC) in China, a new venture encompassing the previously published Beijing Institute of Genomics Data Center. Further papers are grouped in the now-familiar fashion: (i) nucleic acid sequence and structure, transcriptional regulation; (ii) protein sequence and structure; (iii) metabolic and signaling pathways, enzymes and networks; (iv) genomics of viruses, bacteria, protozoa and fungi; (v) genomics of human and model organisms plus comparative genomics; (vi) human genomic variation, diseases and drugs; (vii) plants and (viii) other topics, such as proteomics databases. As ever, the discipline-spanning nature of many modern resources means that readers are encouraged to browse the whole issue. The Nucleic Acids Research online Molecular Biology Database Collection, classifies databases more finely using 15 categories and 41 subcategories, and can be found at http://www.oxfordjournals.org/nar/database/c/.

**Table 1.** Descriptions of new databases in the 2020 NAR Database issue

Database Name	URL	Short description
ABCD	https://web.expasy.org/abcd	AntiBodies chemically defined
Alliance of Genome Resources	https://www.alliancegenome.org	Data united across model organisms
Animal-ImputeDB	http://gong_lab.hzau.edu.cn/Animal_ImputeDB	Reference panels and imputation methods for animal genomes
APAatlas	https://hanlab.uth.edu/apa	Human alternative polyadenylation
BacFITBase	http://www.tartaglialab.com/bacfitbase/	Bacterial genes relevant to host infection
BBCancer	http://bbcancer.renlab.org	Blood-based biomarkers for cancer
CancerGeneNet	https://signor.uniroma2.it/CancerGeneNet	Paths from cancer mutations to ‘Hallmark phenotypes’
CancerTracer	http://cailab.labshare.cn/cancertracer	Tumor heterogeneity in individual patients
CausalDB	http://mulinlab.tmu.edu.cn/causaldb	Predicted causal variants from GWAS
CFEA	www.bio-data.cn/CFEA	Cell-free epigenome atlas
ChlamDB	https://www.chlamdb.ch	Comparative genomics of the Chlamydiae phylum and the PVC superphylum
CRISPRCasdb	https://crisprcas.i2bc.paris-saclay.fr	CRISPR arrays and Cas proteins
DNMIVD	http://www.unimd.org/dnmivd	DNA methylation and cancer
DrLLPS	http://llps.biocuckoo.cn	Liquid–liquid phase separation of proteins
DrugCombDB	http://drugcombdb.denglab.org	Drug combination data
ENdb	http://www.licpathway.net/ENdb	Experimentally supported enhances in human and mouse
EuRBPDB	http://EuRBPDB.syshospital.org	Eukaryotic RNA binding proteins
EWAS Data Hub	http://bigd.big.ac.cn/ewas/datahub	DNA methylation array data and metadata
ExonSkipDB	https://ccsm.uth.edu/ExonSkipDB	Exon skipping
FoldamerDB	http://foldamerdb.ttk.mta.hu	Peptidic foldamers
Gene4Denovo	http://genemed.tech/gene4denovo	Human *de novo* mutations
Genus	http://genus.fuw.edu.pl	Topological characteristics of biomolecular structures
Gephebase	https://www.gephebase.org	Genotype–phenotype relationships in eukaryotes
GMrepo	https://gmrepo.humangut.info	Human gut metagenomics data
gutMDisorder	http://bio-annotation.cn/gutMDisorder	Dysbiosis of the gut microbiota
GWAS Atlas	http://bigd.big.ac.cn/gwas	GWAS studies across seven plants and two animals
KnockTF	http://www.licpathway.net/KnockTF	Gene expression following TF knockdown/knockout
LLPSDB	http://bio-comp.ucas.ac.cn/LLPSDB	Liquid–liquid phase separation of proteins
LnCeVar	http://www.bio-bigdata.net/LnCeVar	lncRNA SNPs affecting ceRNA action
LncTarD	http://bio-bigdata.hrbmu.edu.cn/LncTarD	lncRNA-mediated regulatory mechanisms and disease
MaGenDB	http://magen.whu.edu.cn	Functional genomics hub of Malvaceae eg cotton, cacao
malaria.tools	http://malaria.tools	Plasmodium co-expression and co-function networks
MBKbase	http://www.mbkbase.org	Plant Molecular Breeding Knowledgebase
ncRNA-eQTL	http://ibi.hzau.edu.cn/ncRNA-eQTL	eQTL analysis of ncRNAs
OGRDB	https://ogrdb.airr-community.org	Open germline receptor database
oRNAment	http://rnabiology.ircm.qc.ca/oRNAment	Predicted RBP binding sites in complete transcriptomes
PADS Arsenal	http://bigd.big.ac.cn/padsarsenal	Prokaryotic antiviral defense systems
PathBank	http://www.pathbank.org	Pathways in model organisms
*PGG*.Han	http://www.pgghan.org or https://www.hanchinesegenomes.org	Han Chinese genomes
PhaSepDB	http://db.phasep.pro	Phase separation related proteins
PhaSepPro	http://phasepro.elte.hu	Phase separation related proteins
PhenoModifier	https://www.biosino.org/PhenoModifier	Genetic modifiers of human disorders
PDBe-KB	http://pdbe-kb.org	Structural and functional annotations of the PDB
PmiREN	http://www.pmiren.com	Plant MicroRNA Encyclopedia
Pro-Carb	http://structure.bioc.cam.ac.uk/procarb	Protein–carbohydrate interactions
QTLbase	http://mulinlab.org/qtlbase	Quantitative Trait Loci across human phenotypes
SEAweb	http://sea.ims.bio	Small RNA Expression Atlas
snoDB	http://scottgroup.med.usherbrooke.ca/snoDB	Human snoRNAs
SNP2APA	http://gong_lab.hzau.edu.cn/SNP2APA	SNPs and alternative polyadenylation in cancer
SpatialDB	https://spatialomics.org/SpatialDB/	Spatially resolved transcriptome
SyntDB	http://syntdb.amu.edu.pl	lncRNAs and their evolutionary relationships in primates
TerrestrialMetagenomeDB	https://webapp.ufz.de/tmdb	Terrestrial metagenome metadata
Thera-SAbDab	http://opig.stats.ox.ac.uk/webapps/therasabdab	Therapeutic antibodies
T-psi-C	http://tpsic.igcz.poznan.pl	Experimentally determined tRNA sequences
TSEA-DB	https://bioinfo.uth.edu/TSEADB	Tissue specificity of GWAS traits and phenotypes
VARIDT	https://db.idrblab.org/varidt	Variability of Drug Transporter Database
VDJbase	https://www.VDJbase.org	Genotype and haplotype data from AIRR sequencing
VISDB	https://bioinfo.uth.edu/VISDB/index.php	Virus Integration Site DataBase
YEASTRACT+	http://yeastract-plus.org	Transcription regulation in yeasts

**Table 2.** Updated descriptions of databases most recently published elsewhere

Database Name	URL	Short description
DNAproDB	https://dnaprodb.usc.edu	DNA–protein complex structure analysis
EnhancerAtlas	http://www.enhanceratlas.org/indexv2.php	Enhancers in nine species
GWAS Central	https://www.gwascentral.org	GWAS datasets
MatrisomeDB	http://matrisome.org	Extracellular matrix components
MIBiG	https://mibig.secondarymetabolites.org	Biosynthetic Gene Clusters of Known Function
MirGeneDB	www.mirgenedb.org	Animal miRNA complements
MSDB	http://data.ccmb.res.in/msdb	Microsatellites from all sequenced genomes
Ohnologs	http://ohnologs.curie.fr	Vertebrate ohnologs
PolyASite	http://www.polyasite.unibas.ch	RNA polyadenylation sites
WALTZ-DB	http://waltzdb.switchlab.org	Amyloidogenic peptide sequences

Among the major global centers, the NCBI (1) reports updates across many databases and interfaces. For example, gene searches can now cleverly retrieve orthologs from (subsets of) vertebrates. The EBI paper (2) includes striking figures that illustrate the deep inter-connectedness of its hosted databases, as well as their myriad links to external resources. It also describes a significant new arrival, the BioImage Archive. The paper from the National Genomics Data Center (3) includes descriptions of their rapidly expanding suite of databases, some featured in detail elsewhere in this Issue. They report that their database for raw sequence reads, the Genome Sequence Archive, now occupies more than a petabyte.

In the ‘Nucleic acid databases’ section, major returning databases include miRTarBase (4), the database of experimentally validated miRNA-target interactions, offering a new focus on miRNA regulatory networks; and miRDB (5), the database of predicted miRNA target sites, here reporting an improved predictive algorithm and Gene Ontology-based miRNA function prediction. MirGeneDB (6), appearing in Nucleic Acids Research for the first time, takes an evolutionary approach to manually curate and classify miRNAs, and covers 45 representative metazoa. Elsewhere, LncBase indexes miRNA targets found on ncRNA transcripts, including consideration of sequence variants lying within miRNA binding sites (7). Two returning databases cover the interactions of RNA with other biomolecules more broadly. NPInter (8) covers ncRNA interactions and includes DNA and circRNA partners for the first time, while RNAInter (9), successor to the previously published RAID (10) now holds 8-fold more interaction data than before, and covers a notably wide range of RNAs and interacting molecules. The well-known database of transcription factor binding sites JASPAR (11) provides an Update paper that, interestingly, reports on a collection of unvalidated sites and mechanisms by which the user community can assist in their curation. TFBSshape (12) is another returning database of transcription factor binding sites but focuses on 3D DNA shape, which can change significantly on DNA methylation, as an important contributor to binding specificity. Gene expression data are covered by the well known Expression Atlas (13) which reports a new section for single cell gene expression as well as by the newcomer KnockTF (14) which reports the impact on expression of transcription factor knockout or knockdown experiments. Another new database SpatialDB (15) provides spatially resolved transcriptome data across 10 experimental methods and five species. With the arrival of snoDB (16), human snoRNA molecules—with important roles in directing RNA post-transcriptional modifications but increasingly suspected of a range of other functions—gain a new dedicated database. Finally, two new databases focus on alternative polyadenylation in human cells. APAatlas (17) majors on the tissue specificity of the process while SNP2APA (18) considers how the impact of SNPs on alternative polyadenylation links to cancer.

Two of the issue's Breakthrough articles are found in the section on protein sequence and structure databases. The ABCD (AntiBodies Chemically Defined) database (19) curates information about antibodies and their antigens, linking out to standard databases from each. One of the main drivers for the establishment of the database was experimental reproducibility since, as the authors note, poorly defined or batch-variable antibodies are a major issue (20). ABCD therefore assigns a unique identifier to each antibody sequence (represented by V_L_ and V_H_ chains) with a known antigen. With an eye to the sustainability of this curated database, and acknowledging the time-consuming nature of literature mining, the authors encourage submission of entries directly by colleagues in the field. Another valuable new database relating to antibodies Thera-SAbDab (21) links therapeutic antibody or nanobody sequences recognized by the World Health Organisation to entries in the authors’ structural antibody database SAbDab (22) for similar or identical proteins.

The second breakthrough article reports the return of the SCOP (Structural Classification of Proteins) resource after a number of years (23). The new iteration of the database adopts a simplified version of the schema published in prototype form in 2013 (24). At that time the original authors broke away from their original conception of a purely hierarchical database, although their original structure has since seen continued and very valuable maintenance by the SCOPe team (25). SCOPe continues to be highly used but there will be strong interest among the structural bioinformatics community in the new, more flexible relationships allowed by SCOP in 2020. The new version also includes definitions of intrinsically unstructured protein regions. These and the traditional folded domains are now placed into four protein types: soluble, membrane, fibrous and intrinsically disordered. While the database remains largely hierarchical, the authors illustrate the non-hierarchical relationships now usefully captured by the new schema. Another foundational resource in protein bioinformatics, the Conserved Domain Database (26), reports an update of its own hierarchical protein sequence family annotation framework. Elsewhere the major news is the arrival of no fewer than four databases devoted to proteins involved in liquid–liquid phase separation (27–30). These proteins form the basis of membraneless organelles/condensates (31), which are found in various cellular compartments and whose dysfunction is increasingly linked to disease (32). Better known classes of protein are covered in the returning DisProt (33), for protein intrinsic disorder and WALTZ-DB (34) for amyloidogenic protein sequences. Elsewhere the regular PDBe update (35) reports on improved search methods, enhanced links to other databases for eg RNA molecules, and better identification of cofactors. A new associated database PDBe-KB (36) offers annotations of PDB deposits from an impressive array of 18 partner resources, many familiar to readers of the NAR Database and Webserver Issues. With notably stylish and intuitive presentation, PDBe-KB pages offer an efficient way to browse functional features of a protein structure of interest.

A number of major pathway databases contribute updated papers to the metabolic and signaling section. They include Pathway Commons (37) which integrates data from a large number of pathway and interaction databases. The developers report, however, that few of them are currently funded and so, in order to address the rapidly growing literature, a curation support tool is planned to allow authors to submit summaries of their new papers for curation. Reactome reports its own Update (38) which also reports on efforts to engage the community in contributing to the resource, and includes a striking new Voronoi diagram browser to visualize pathways. A major new arrival in the area is PathBank (39) which aims to comprehensively catalog both metabolic and signaling pathways in model organisms. It too seeks community input and majors on pathway coverage and options for search, visualization and download of data. Metabolic and signaling models are the focus of two returning databases. BIGG Models (40) continues to expand its content of genome-scale metabolic models, including multi-strain models for the first time, and now links to a model validation tool. An updated paper from BioModels (41) reports content totalling around 2000 models. These are subject to targeted curation and the authors show that users strongly prefer curated models to uncurated. Future plans include acceptance of computer-submitted models and enabling storage and dissemination of multi-scale models. Signaling is the focus of two databases, MiST (42) returning after a decade's absence with a new interface to its cataloging of microbial signaling systems, proteins and domains, and SIGNOR (43) reporting a near doubling in size of its graph representations of information flow in eukaryotic cells, especially in human. The widely used MiBiG database (44) of biosynthetic gene clusters arrives in Nucleic Acids Research, reporting recent expansion from both community contributions and in-house efforts, and with improved links out to small molecule databases. In the same area, IMG/ABC (45) returns with v.5.0, exploiting recent improvements in the antiSMASH genome mining pipeline (46), and offering higher quality data covering more types of cluster. Finally, MetaboLights (47) reports an update after seven years away that shows a rapidly increasing submission rate. The paper includes a strong focus on the user experience, reporting not only improvements to the submission pipeline, but also a website redesign driven by usability testing.

The microbial genomics section contains a pair of returning resources that focus on antimicrobial resistance. The very popular CARD database (48) reports on the challenges of coping with processing 5000 papers a year in the area of antimicrobial resistance and, among other innovations, now includes computationally predicted resistome data. The MEGARes Update (49) shows how the inclusion of metal and biocide resistance determinants contributes to a near-doubling of size. An improved pipeline for computational annotation of the resistome of metagenomic samples is also described. The new database PADS Arsenal (50) covers Prokaryotic Antiviral Defense Systems of 18 different kinds across more than 30 000 prokaryotes. An impressive variety of visualizations and analytical tools are offered. CRISPRCasdb (51) is a new database which includes both CRISP arrays and Cas proteins and assigns system type and sub-type. Two general comparative genomics resources contribute Update articles. MicroScope (52) reports a number of new tools to annotate genes and genomic regions, aimed at prediction of properties such as function, essentiality, virulence and antibiotic resistance. The PATRIC Bioinformatics Resource Center paper (53) reports that 250 000 genomes are now covered. In accord with their focus on pathogens, antimicrobial resistance is a major topic, but they also report new analytical and visualization tools. Pathogen–host interactions are covered by both PHI-Base (54) which reports a big expansion and increased use of its annotations by other databases; and the significant new arrival BacFITBase (55) which applies a standardized reprocessing to published data to enable assessment of how important genes from 15 pathogenic bacteria are to infection of five vertebrate hosts. The major metagenomics platform MGnify (55) (formerly EBI Metagenomics) has an update that describes improvements to its assembly and analysis pipeline, the introduction of a new system of unique and stable accession numbers, and the easy availability of a rapidly expanding MGnify protein sequence database which is usefully non-redundant with UniProtKB.

In the next section, there is again a strong presence from model organism (MO) databases. The Issue's third Breakthrough Article describes the new Alliance of Genome Resources (56). The Alliance is an important strategic effort to bring together the fruits of all the individual annotator expertise employed at the contributing MO resources. At their portal a user can search for a gene from a favorite MO and receive expression, phenotype and orthology information from across all the contributing MOs. Ribbon representations show, for example, Gene Ontology and disease associations across orthologous genes. One particularly clever feature is the automated gene description which produces a human-readable summary optimally summarizing the ontology terms relating to a gene in a text of a given length. Sensibly, the Alliance authors are seeking to share data models and computational pipelines across resources to facilitate the future sustainable integration of current and future database partners. The Alliance lies in similar territory to a previous Breakthrough paper recipient, the Monarch Initiative for linking genes, variants, genotypes, phenotypes and diseases across species. Here it reports (57) more covered species, more data sources, a new disease ontology and a new website. One particularly nifty new feature is the text annotation widget which marks up a tranche of text, such as a paper abstract, with links to ontologies that can be further explored. Among the Alliance's members contributing Updates are the Rat Genome Database (58), which fittingly celebrates its 20th anniversary in the Year of the Rat, the Saccharomyces Genome Database (59) and Wormbase (60). Returning after a decade's absence, SilkDB reports 3.0 (61) with a higher quality genome assembly, pangenome data to compare genome variants, tissue-level transcriptomics data and an impressively wide range of data representations. The cornerstone project Ensembl offers its usual Update (62) describing very significant improvements, including 94 new vertebrate genomes, new tools to annotate and visualize variants and better resources for epigenomic data. It is joined by its companion database Ensembl Genomes (63) for non-vertebrate genomics. This latter paper breaks the news that both will be accessible from a single website during 2020 as a reflection of the greater integration of the two projects that is seen as necessary to optimally process and display the results of megascale sequencing projects like the Earth BioGenome Project (64). Elsewhere, Ohnologs reports its v2 release (65). Whole genome duplication has played a significant role in vertebrate evolution especially, and the Ohnologs database focuses on those genes that are retained after duplication.

The large number of databases in human genomic variation, diseases and drugs include a number of returning major players. The big news from the IUPHAR/BPS Guide to Pharmacology (66) is a new extension, the IUPHAR/MMV Guide to Malaria Pharmacology, a joint initiative with the Medicines for Malaria Venture and accessible through its own URL (www.guidetomalariapharmacology.org). The heavily used IPD-IMGT/HLA database (67) reports on continued strong growth in its content of named HLA alleles, while an update on the popular Allele Frequency Net Database (68), covering polymorphisms of several immune-related genes, describes a new categorization of HLA data into gold, silver and bronze quality categories revealing some global disparities in sampling. An important new arrival in the immunoinformatics field is VDJbase (69), designed specifically to store genotype and haplotype data for the results of adaptive immune receptor repertoire sequencing (AIRR-seq). Researchers are invited to submit their own datasets which will be validated, processed and deposited in the database. The returning database VDJdb (70) focuses on T-cell receptor sequences and their cognate antigens. It reports huge recent growth and an interface to assist with analysis of large datasets such as those deriving from AIRR-seq. Another significant new arrival in the area is OGRDB (71) a database of immune receptor germline sequences aiming to provide germline gene reference sets for proper interpretation of AIRR-seq data. Several databases that are either new or featuring here for the first time explore Genome-Wide Association Study data and its ability to pinpoint the genomic variability underlying traits and diseases. The paper from GWAS Central (72) describes how it covers nearly 4000 human studies, and plans to map between human and mouse data as the mouse can be used to validate human GWAS findings. The new GWAS Atlas (73), in contrast, focuses on plants and domesticated animals. Another new resource CausalDB (74) focuses on applying fine-mapping tools to try to sift true causal variants from GWAS data while TSEA-DB (75) offers an interesting tissue-specific view of GWAS traits and phenotypes. All efforts to link genome variation to phenotype benefit from complete and balanced representations of species diversity and so the arrival of the *PGG*.Han database (76) is welcome, focusing as it does on the Han Chinese, a group hitherto under-represented in population genomics data. Among resources for drug design it is worth highlighting the popular Therapeutic Target Database (77) which reports a number of new features including target regulators (both miRNA and TF), target-interacting proteins and information targets of patented therapeutic agents. TDR Targets (78) focuses particularly on drug design to address neglected tropical diseases and incorporates new data into a network representation to allow, for example, whole-genome target prioritization and exploration of drug repurposing. It also contributes a stylish cover image to this Issue. Two other heavily used databases, ClinVar (79) and DisGeNET (80) also contribute Updates, each featuring expanded content and a new web interface. As ever, cancer databases are well-represented with new contributions to the field including CancerTracer (81), a resource for studying and intrapatient tumor heterogeneity that features data from 1500 patients, including patient-specific tumor phylogenetic trees, and DNMIVD (82) which has a wide range of functions regarding links between DNA methylation and cancer.

A major returning plant database is PlantRegMap (83) which exploits the information in its associated database of plant transcription factor binding sites PlantTFDB to help predict the functional regulatory maps of dozens of plants. A single paper (84) reports updates to both AraPheno and AraGWAS Catalog databases that include RNA-Seq and knockout mutation data for the model organism *Arabidopsis thaliana*. The Malvaceae, which include such important crops as cotton and cacao gain their own dedicated functional genomics database MaGenDB (85) which includes over 300 diverse omics datasets and a customized genome browser. Also very much orientated toward agricultural purposes is MBKbase (86), a plant molecular breeding knowledgebase. It includes germplasm information and genomic, population sequencing, phenotypic and gene expression data.

The final section, with databases not easily lying within the earlier categories, contains the usual intriguing and eclectic mix. Proteomics research is covered by two major returning databases. ProteomeXchange (87) covers changes made at each of its member organizations, now—with the addition of iProX and Panorama Public—totaling six. ProteomicsDB (88)—which also encompasses diverse data such as gene expression and, in the new release, protein turnover information—now supports organisms other than the original human, with *A. thaliana* leading the way. The popular MatrisomeDB database (89) covering proteomics of the extracellular matrix is appearing here for the first time with a new version tripling the number of datasets of the original. Elsewhere, FoldamerDB (90) addresses peptidic foldamers, non-natural oligomers with defined solution structures that mimic the behavior of natural macromolecules and have potential in areas as diverse as antimicrobial therapy and materials science. Finally, the Genus database (91) contains calculations relating to the genus, a topological property, of all protein and RNA molecules in the PDB, also allowing analysis of structures uploaded by users.

## NAR ONLINE MOLECULAR BIOLOGY DATABASE COLLECTION

For this 27th release of the NAR online Molecular Database Collection (as usual freely available at http://www.oxfordjournals.org/nar/database/c/), in our ongoing process to provide and up-to-date resource, over the last year we have updated 305 entries, added 65 new resources and deleted 125 discontinued databases, bringing the total collection to 1637 databases.

This is a never ending task, as once this publication sees the light, there may be listed resources going down which we will not detect until our next scheduled review during the year, at which point those database owners will be informed. Ignored requests or lack of action will result in databases listed as obsolete being deleted in future updates of the collection. We encourage authors to submit their updates to XMF at xose.m.fernandez@gmail.com in plain text, ideally according to the template found in http://www.oxfordjournals.org/nar/database/summary/1.
